# Genetic and Phenotypic Characterization of Indole-Producing Isolates of *Pseudomonas syringae* pv. *actinidiae* Obtained From Chilean Kiwifruit Orchards

**DOI:** 10.3389/fmicb.2018.01907

**Published:** 2018-08-22

**Authors:** Oriana Flores, Camila Prince, Mauricio Nuñez, Alejandro Vallejos, Claudia Mardones, Carolina Yañez, Ximena Besoain, Roberto Bastías

**Affiliations:** ^1^Laboratorio de Microbiología, Instituto de Biología, Facultad de Ciencias, Pontificia Universidad Católica de Valparaíso, Valparaíso, Chile; ^2^Departamento de Análisis Instrumental, Facultad de Farmacia, Universidad de Concepción, Concepción, Chile; ^3^Laboratorio de Fitopatología, Escuela de Agronomía, Pontificia Universidad Católica de Valparaíso, Valparaíso, Chile

**Keywords:** *Pseudomonas syringae* pv. *actinidiae*, Psa Biovar 3 (Psa-V), MLSA, kiwifruit, IAA, IAA production, indoleacetic acid lysine, IAA-L

## Abstract

In recent years, Chilean kiwifruit production has been affected by the phytopathogen *Pseudomonas syringae* pv. *actinidiae* (Psa), which has caused losses to the industry. In this study, we report the genotypic and phenotypic characterization of 18 Psa isolates obtained from Chilean kiwifruits orchards between 2012 and 2016 from different geographic origins. Genetic analysis by multilocus sequence analysis (MLSA) using four housekeeping genes (*gyrB, rpoD, gltA*, and *gapA*) and the identification of type III effector genes suggest that the Chilean Psa isolates belong to the Psa Biovar 3 cluster. All of the isolates were highly homogenous in regard to their phenotypic characteristics. None of the isolates were able to form biofilms over solid plastic surfaces. However, all of the isolates formed cellular aggregates in the air–liquid interface. All of the isolates, except for Psa 889, demonstrated swimming motility, while only isolate Psa 510 demonstrated swarming motility. The biochemical profiles of the isolates revealed differences in 22% of the tests in at least one Psa isolate when analyzed with the BIOLOG system. Interestingly, all of the isolates were able to produce indole using a tryptophan-dependent pathway. PCR analysis revealed the presence of the genes *aldA*/*aldB* and *iaaL*/*matE*, which are associated with the production of indole-3-acetic acid (IAA) and indole-3-acetyl-3-L-lysine (IAA-Lys), respectively, in *P. syringae*. In addition, IAA was detected in the cell free supernatant of a representative Chilean Psa strain. This work represents the most extensive analysis in terms of the time and geographic origin of Chilean Psa isolates. To our knowledge, this is the first report of Psa being able to produce IAA. Further studies are needed to determine the potential role of IAA in the virulence of Psa during kiwifruit infections and whether this feature is observed in other Psa biovars.

## Introduction

*Pseudomonas syringae* pv. *actinidiae* (Psa) is the causal agent of bacterial canker in *Actinidia deliciosa* and *Actinidia chinensis* that has caused severe losses in all of the major areas of kiwifruit cultivation, including Italy, China, New Zealand, and Chile (Scortichini et al., 2012; Ferrante and Scortichini, 2015). This bacterium infects host plants by entering natural openings and wounds, moving inside the plant, and promoting the appearance of necrotic leaf spots, red exudate production, and canker and necrosis in the trunk. In the late stages of the infection, the plants wilt and desiccate which leads to the death of the kiwifruit vine (Vanneste et al., 2012; Cellini et al., 2014). Since its identification in Japan in 1984, successive outbreaks of Psa have been observed worldwide, and therefore it is now considered to be a pandemic phytopathogen (Scortichini et al., 2012; McCann et al., 2017). Comparative analysis using multilocus sequence analysis (MLSA), the detection of type III secretion system effector genes and phytotoxins (phaseolotoxin or coronatine) in Psa isolates from different geographic origins have revealed the existence of five clusters of biovars (Marcelletti et al., 2011; Ciarroni et al., 2015; Ferrante and Scortichini, 2015; Fujikawa and Sawada, 2016; McCann et al., 2017): biovar 1, comprising Japanese strains which are able to produce phaseolotoxin; biovar 2, including only South Korean strains which produce coronatine; biovar 3 or Psa-V, which includes the most virulent strains that are characterized by not producing phytotoxins and were first isolated in Italy (2008–2009) and have been subsequently reported to cause outbreaks in different countries (Butler et al., 2013; Ciarroni et al., 2015; Cunty et al., 2015a); biovar 4, contain strains with low virulence and was recently proposed to be a new pathovar called *P. syringae* pv. *actinidifoliorum* (Psaf) (Abelleira et al., 2015; Cunty et al., 2015b); and finally, biovar 5 with Japanese strains isolated in 2012 which do not produce phytotoxins. Recently, a potential new biovar was described in Japan, which produces both phaseolotoxin and coronatine (Fujikawa and Sawada, 2016).

The genetic analysis of the Psa biovars described a set of genes that participate in distinct phases of kiwifruit infection and niche colonization, both outside and inside of the host plant. These genes are related to bacterial motility, biofilm formation, copper and antibiotic resistance, siderophore production, and the degradation of lignin (Marcelletti et al., 2011; Scortichini et al., 2012; Ghods et al., 2015; Gao et al., 2016; Colombi et al., 2017; Patel et al., 2017). However, the mechanisms that determine infection and the interactions between Psa with the kiwifruit plant remain unknown. The production of the phytohormone indole-3-acetic acid (IAA) is another virulence factor that has been described in *Pseudomonas savastanoi* and *P. syringae* pathovars. This compound can perturb the regulation of the hormone balance in the plant and increase its susceptibility to infection (Glickmann et al., 1998; Cerboneschi et al., 2016). IAA production using the indole-3-acetamide (IAM) pathway is the most common mechanism in phytopathogenic bacteria, including *P. syringae*, and has mostly been characterized in *P. savastanoi* pv. *savastanoi* (Psav) (Baltrus et al., 2011; Aragón et al., 2014) where IAA biosynthesis begins from L-tryptophan (Trp) and involves the activity of the enzymes tryptophan-2-monooxygenase (IaaM) and IAM hydrolase (IaaH) encoded by the *iaaM* and *iaaH* genes, respectively. However, in other *P. syringae* pathovars, the IAA production involves other genes that lack homology to *iaaM* and *iaaH* (Glickmann et al., 1998), and recently aldehyde dehydrogenase family proteins encoded by genes *aldA* and *aldB*, were associated with IAA synthesis in *P. syringae* pv. *tomato* (McClerklin et al., 2018). For instance, *P. savastanoi* pv. *nerii* can conjugate IAA to the amino acid lysine producing indole-3-acetyl-3-L-lysine (IAA-Lys) due to the action of the enzyme IAA-Lys ligase encoded by the *iaaL* gene (Cerboneschi et al., 2016). This gene has been found in several *P. syringae* pathovars where it is arranged in synteny with the gene *matE*, which encodes a putative MATE family transporter, and has been implicated in the fitness and virulence of *P. syringae* pv. *tomato* (*Pst*) in tomato plants (Glickmann et al., 1998; Castillo-Lizardo et al., 2015).

*Pseudomonas syringae* pv. *actinidiae* was first reported in Chile in 2010 following its isolation from kiwifruit orchards in the Maule Region, and since 2011, it has been considered to be a pest under the official control of the Agricultural and Livestock Service (SAG) of the Government of Chile (McCann et al., 2013). Previous studies included classifying the first Chilean Psa isolates in biovar 3 together with strains from China, Europe, and New Zealand (Butler et al., 2013; McCann et al., 2013; Cunty et al., 2015a). However, the scope of these studies was limited by the number of Chilean strains. In this study, we report the genotypic and phenotypic characterization of Chilean Psa isolates obtained between 2012 and 2016 from the regions that accumulate more than 80% of the Psa-positive orchards in Chile. In addition, we show the first evidence of Psa strains producing IAA.

## Materials and Methods

### Bacterial Strains and Culture Conditions

Chilean Psa isolates are listed in **Table 1** and were obtained from the SAG from kiwi orchards of different geographic areas in the central-south of Chile in 2012, 2013, and 2016. *P. syringae* pv. *tomato* DC3000 was provided by Dr. Paula Salinas of the Universidad Santo Tomás (Santiago, Chile). *Escherichia coli* DH5α, *E. coli* K12, *Pseudomonas aeruginosa* PAO1, *Azospirillum brasilense* SP7, *Salmonella bongori* X9617, and *Cupriavidus metallidurans* CH34 were obtained from the bacterial collection of the Laboratory of Microbiology of the Pontificia Universidad Católica de Valparaíso (PUCV). *Pseudomonas antarctica* S63 (Vásquez-Ponce et al., 2018) was provided by Dr. Jorge Olivares from the PUCV. The bacteria were grown at 25°C in Luria-Bertani (LB) medium except when another medium is specified. Growth curve were performed in 96 multi-well plates at 25°C during 30 h in a microplate spectrophotometer Infinite^®^ M200 NanoQuant (TECAN). Optical density (OD_600__nm_) was determined each 30 min. All curves were performed in biological triplicates.

**Table 1:** Chilean Psa isolates used in this work.

Isolate^∗^	Place of collection (coordinates)	Year
Psa 743	Linares, Maule (35°52′54.4^′′^S 71°35′23.4^′′^W)	2012
Psa 889	Retiro, Maule (35°54′47.4^′′^S 71°44′20.3^′′^W)	2012
Psa 817	Chillán, Bío Bío (36°40′43.6^′′^S 71°54′13.7^′′^W)	2012
Psa 381	Molina, Maule (35°07′40.2^′′^S 71°15′05.1^′′^W)	2013
Psa 510	Retiro, Maule (36°08′53.3^′′^S 71°44′54.3^′′^W)	2013
Psa 771	Retiro, Maule (36°01′28.5^′′^S 71°44′40.0^′′^W)	2013
Psa 784	Retiro, Maule (36°01′16.9^′′^S 71°44′18.1^′′^W)	2013
Psa 394	Colbún, Maule (35°43′17.5^′′^S 71°28′56.4^′′^W)	2013
Psa 387	Yerbas Buenas, Maule (35°59′12.0^′′^S 71°34′45.2^′′^W)	2013
Psa 882	Molina, Maule (35°07′40.2^′′^S 71°15′05.1^′′^W)	2013
Psa 144	Molina, Maule (35°07′39.8^′′^S 71°15′03.2^′′^W)	2013
Psa 598	San Carlos, Bío Bío (36°30′18.6^′′^S 71°51′50.2^′′^W)	2013
Psa 386	San Ignacio, Bío Bío (36°52′59.9^′′^S 72°08′07.0^′′^W)	2013
Psa 159	San Ignacio, Bío Bío (36°48′38.6^′′^S 72°06′12.6^′′^W)	2013
Psa 189	San Ignacio, Bío Bío (36°49′25.2^′′^S 72°06′06.6^′′^W)	2013
Psa 129	San Ignacio, Bío Bío (36°48′38.6^′′^S 72°06′12.6^′′^W)	2013
Psa 137	San Nicolás, Bío Bío (36°32′39.1^′′^S 72°10′16.6^′′^W)	2015
Psa 233	Molina, Maule (35°03′09.5^′′^S 71°14′57.1^′′^W)	2016


*^∗^Accession number of the nucleotide sequences of the *gapA, gltA, gyr*, and *rpoD* genes added to GenBank (NCBI) are included in **Supplementary Table S3***.

### Molecular Identification and Characterization of the Psa Isolates

*Pseudomonas syringae* pv. *actinidiae* strain molecular identification was performed using RG-PCR and duplex-PCR as previously described (Rees-George et al., 2010; Gallelli et al., 2011). For RG-PCR, specific primers were used to amplify the internal transcribed spacer (ITS) between the 16S and 23S rRNA sequences, and for duplex-PCR, specific primers against *ompP1* (Outer Membrane Protein P1) and *avrD1* (effector) genes were used. All 18 isolates amplified produced bands of the expected size (**Supplementary Figure S1**). In addition, the identity of these isolates was also confirmed by partial 16S rDNA sequences. For genomic DNA isolation, the bacteria were grown in LB media for 16 h until the stationary phase. Total genomic DNA was extracted using a Wizard^®^ Genomic DNA Purification Kit (Promega) according to the manufacturer’s instructions. The DNA concentration was determined using MaestroNano MN-913 (Maestrogen, Inc.). For the molecular identification of the type III effector genes, the reference genome of Psa ICMP 18884 biovar 3 strain (GenBank accession number: NZ_CP011972.2) (Templeton et al., 2015) and contigs of the Chilean Psa genomes, ICMP 19439 (ANJM00000000.1) and ICMP 19455 (ANJK00000000.1), available in GenBank (NCBI) were used to design specific primers for the PCRs. Comparative sequence analysis was performed using the Geneious R11 software (Kearse et al., 2012). The amplicons of effector genes obtained from strain Psa 743 were purified using an E.Z.N.A.^®^ Cycle Pure Kit (Omega Bio-Tek, Inc.) and sequenced using the Sanger method by Macrogen, Inc. (South Korea). The quality and assembly of the sequences were analyzed using Geneious R11 software, which were compared with the NCBI database using BLASTN and BLASTX to identify the genes. Primers and annealing temperatures used in the PCRs are listed in **Supplementary Table S1**. In all cases, PCR was performed on a SureCycler 8800 Thermal Cycler (Agilent Technologies) using SapphireAmp Fast PCR Master Mix (Takara Bio) according to the manufacturer’s instructions. PCR products were separated using electrophoresis in agarose gel (1.5% agarose in 1× buffer TAE) stained with GelRed^TM^ (Biotium), and the bands were visualized under UV light. PCRs were performed in triplicate. The genomic DNA of *P. syringae* pv. *tomato* DC3000 and *E. coli* DH5α were used as the control reactions. The sequences of the effector genes of a selected strain (Psa 743) were deposited in GenBank (NCBI), and the accession numbers are listed in **Supplementary Table S2**.

### Phylogenetic Analysis by MLSA

The *gapA, gltA, gyrB*, and *rpoD* genes, encoding glyceraldehyde-3-phosphate dehydrogenase, citrate synthase, DNA gyrase B, and sigma factor 70, respectively, were amplified from the genomic DNA of Psa isolates using the primers listed in **Supplementary Table S1** as previously described (Ferrante and Scortichini, 2010). PCR was performed in triplicate using a SureCycler 8800 Thermal Cycler (Agilent Technologies) with *GoTaq* G2 Flexi polymerase (Fermentas) according to the manufacturer’s instructions. The PCR products were visualized using electrophoresis in agarose gels and purified using an E.Z.N.A.^®^ Gel Extraction Kit (Omega Bio-Tek, Inc.). The automated sequencing of the amplicons was performed by Macrogen, Inc. (South Korea), and the sequences were analyzed using the Geneious R9 software package (Biomatters Limited) (Kearse et al., 2012). The nucleotide sequences of the *gapA, gltA, gyr*, and *rpoD* genes of Chilean Psa strains were added to GenBank (NCBI) and are listed in **Supplementary Table S3**. The sequences of other Psa biovars available in GenBank (NCBI) were included in the analysis and are listed in **Supplementary Table S4**. In addition, sequences of *P. syringae* pv. *tomato* strain DC3000 were included: *gapA* (AE016853.1:1415258-1416259), *cts* (AE016853.1:2414332-2415621), *gyrB* (AE016853.1:4147-6564), and *rpoD* (AE016853.1:588846-590696) (Buell et al., 2003). The sequences of each locus were aligned using the CLUSTALW included in the MEGA7 software (Kumar et al., 2016). A dendrogram from four-locus concatenated sequences was generated using neighbor-joining (UPGMA) and 1,000 bootstrap iterations.

### Biochemical Characterization

The bacteria were streaked out from a -80°C stock onto LB plates and incubated at 25°C for 48 h. Biochemical patterns were determined using the Biolog GEN III MicroPlate^TM^ system (Biolog^TM^, United States) according to the manufacturer’s instructions. BIOLOG plates were read in an Infinite M200 PRO plate reader, TECAN. Reactions were considered positive if the OD_590__nm_ was greater than 50% of the positive control (∼0.7). Reactions indistinguishable from the negative control and with an OD_590__nm_ below 25% of the positive control (∼0.35) were considered to be negative. Reactions between these two parameters were considered borderline.

### Determination of Streptomycin and Copper Susceptibility

The copper and streptomycin susceptibility was determined using the broth microdilution method (Biebl and Pfennig, 1978; Mergeay et al., 1985). Bacterial strains were grown in Tris minimal (for the copper assay) or Mueller–Hinton (for the streptomycin assay) media during 18 h, and the optical density at 600 nm (OD_600__nm_) was adjusted to 0.7. For the copper susceptibility assays, 10 μL of each bacterial culture were inoculated in Tris minimal agar media (1.5% agar) supplemented with the corresponding copper sulfate concentration (0, 75, 100, 125, 150, 175, 200, 225, 250, 275, and 300 μg/mL). To assess the streptomycin susceptibility, bacterial strains were inoculated in Mueller–Hinton agar media supplemented with the corresponding antibiotic concentration (0, 3.9, 7.8, 15.7, 31.25, 62.5, 125, 250, 500, 1,000, and 2,000 μg/mL). Plates were incubated for 5 days at 25°C, and the bacterial growth was observed. *C. metallidurans* CH34 and *P. antarctica* S63 were used as experimental controls (von Rozycki and Nies, 2009; Vásquez-Ponce et al., 2018). All experiments were performed in biological and technical triplicates.

### Biofilm Production

Microtiter plate biofilm production was performed and adapted as previously described (Merritt et al., 2011; O’Toole, 2011; Ueda and Saneoka, 2015). Briefly, overnight bacterial cultures were adjusted to an optical density of 0.1 (OD_600__nm_) and diluted 10-fold. Aliquots (100 μL) of the dilution were added to each well (96-well microtiter plates), and the plates were incubated for 7 days at 25°C. After incubation, the liquid supernatant was removed and the plates were washed with distilled water. The wells were stained with 0.1% violet crystal solution, and the biofilm was solubilized with a 30% acetic acid solution. The biofilm production was quantified spectrophotometrically (550 nm) in a Tecan Infinite M200^®^ microplate reader. For the air–liquid interface biofilm assay, 1 mL of the bacterial dilution was added to each well (12-well plates), and the plates were incubated at 25°C for 96 h. Surface biofilm formation was monitored and photo documented every 24 h. All of the experiments were performed in biological and technical triplicates, and *P. aeruginosa* PAO1 was used as the positive control (Ghafoor et al., 2011).

### Bacterial Motility Assay

Motility assays were adapted for the Psa assays as described by Hosseinidoust et al. (2013). Swimming motility assays were performed by inoculating 2 μL of stationary-phase bacterial culture (OD_600__nm_∼1.3) into the center of 0.3% LB agar plates. Swarming motility assays were performed utilizing the same procedure except that 0.5% LB agar plates were used. The zone sizes were measured after incubation at 30°C for 72 h. The assays were performed in biological and technical triplicates. *E. coli* K12 was used as the experimental control (Swiecicki et al., 2013). Statistical analysis was performed using one-way ANOVA and Dunnett’s multiple comparison test with *p* ≤ 0.05.

### Indole Production and Identification of IAA Pathway Genes

The indole production was determined using Salkowski’s method as previously described (Mazzola and White, 1994; Mohite, 2013). Briefly, each strain was grown in LB media supplemented with Trp (2 g/L) and incubated at 25°C for 24 h. After incubation, the bacterial density was measured (OD_600__nm_), and the cultures were centrifuged at 10,000 rpm for 10 min. Cell-free supernatants were mixed with 0.5 mL of Salkowski’s reagent (12 g of FeCl_3_ per liter in 7.9 M H_2_SO_4_). The mixture was incubated for 30 min at room temperature in the dark, and the absorbance at 530 nm was determined. The concentration of indole in each sample was determined using a standard curve of indoleacetic acid (Sigma) (0–30 μg/mL) (**Supplementary Figure S3**). IAA concentrations were normalized to the cell density. *A. brasilense* SP7 (Bar and Okon, 1993) and *S. bongori* X9617 (De La Rosa Fraile et al., 1980) strains were used as experimental positive and negative controls, respectively. All of the analyses were performed in biological and technical triplicates. Statistical analysis was performed using one-way ANOVA and Dunnett’s multiple comparison test with *p* ≤ 0.05. The detection of *iaaL, matE, iaaH, iaaM, aldA*, and *aldB* genes in the Chilean Psa isolates was performed using specific primers designed on the basis of conserved regions from the sequences of different *P. syringae* pathovars (**Supplementary Table S5**). The primers designed are listed in **Supplementary Table S1**. PCRs were performed on a SureCycler 8800 Thermal Cycler (Agilent Technologies) using a SapphireAmp Fast PCR Master Mix (Takara Bio) according to the manufacturer’s instructions. The PCR conditions were as follows: 5 min at 95°C, followed by 35 cycles of 30 s at 95°C, 30 s at the annealing temperature (**Supplementary Table S1**), 2 min at 72°C, and a final elongation step of 5 min at 72°. Sanger automated sequencing of the amplicons from Psa 743, Psa 598, and Psa 889 was performed by Macrogen, Inc. (South Korea). The sequences were compared with those in the NCBI database using BLASTN and BLASTX for gene identification. The sequences obtained were deposited in GenBank (NCBI), and the accession numbers are listed in **Supplementary Table S2**.

### LC-ESI-MS/MS Analysis

To detect IAA, Psa strain 743 was grown in minimal media (4.5 g/L KH_2_PO_4_, 10.5 g/L K_2_HPO_4_, 1 g/L (NH_4_)_2_SO_4_, and 0.5 g/L sodium citrate) supplemented with Trp (2 g/L) and incubated at 25°C for 72 h. After incubation, the bacterial density was measured (OD_600__nm_), and the cultures were centrifuged at 10,000 rpm for 10 min. The supernatant was filtered (0.22 μm). Methanol and acetic acid were added to the cell-free supernatant at a final concentration of 10 and 0.05%, respectively, and then filtered through a PVDF filter (0.22 μm). At the end, the sample was subjected to LC-ESI-MS/MS analysis using indoleacetic acid and lysine (Sigma) as standards. The analysis was performed using a Shimadzu Nexera HPLC system coupled to a 3200Q TRAP mass spectrometer equipped with a turbo ion spray interface (Applied Biosystems/MDS Sciex, ON, Canada). A Kinetex C18 core shell column (150 mm × 4.6 mm i.d.; 2.6 μm particle size; Kinetex, Phenomenex) protected by a C18 UHPLC Ultra column guard (0.5 μm Porosity × 4, 6 mm. i.d., Phenomenex, United States) was used. The elution gradient was adapted from Matsuda et al. (2005) and consisted of a mixture of methanol:water containing 0.05% acetic acid (methanol gradient: 10–90% in 13 min; 95% from 13.1 to 28 min) at a flow rate of 0.4 mL/min and a column temperature of 30°C. MS was conducted in the positive ion mode during the following conditions: curtain gas (CUR), 10 psi; collision activated dissociation (CAD), medium; ion spray voltage (IS), 4500 V; nebulizer gas (Gas1), 60 psi; turbo gas (Gas2), 40 psi; temperature (TEM), 400°C. The detection was performed using multiple reaction monitoring (MRM). The data obtained were processed using Analyst 1.3 software (Applied Biosystems).

## Results

### Phylogenetic Analysis and Molecular Characterization of the Chilean Psa Isolates

**FIGURE 1:**
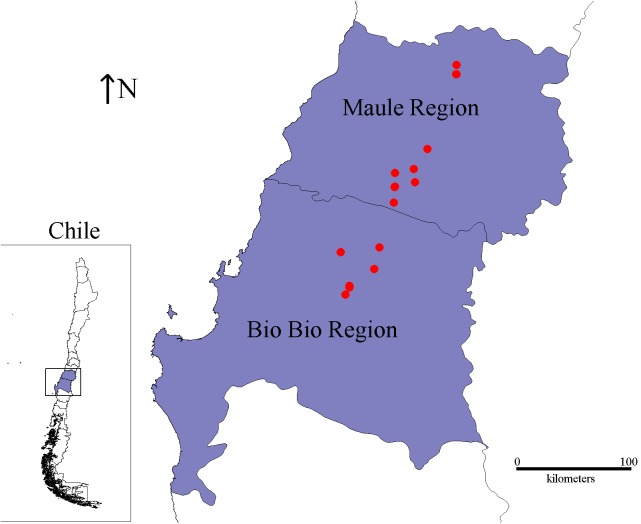
Geographic localization of the Chilean Psa strains. The map shows the localization of the Maule and Bío Bío regions. The red spots represent the exact location from where the Chilean Psa strains were isolated.

The 18 Chilean Psa isolates used in this study were collected from kiwi plants with canker disease symptoms by the SAG. These isolates were obtained between 2012 and 2016 from orchards in central-south Chile (Bío Bío and Maule Regions) that is the site of the vast majority of kiwifruit production in the country (Oficina de Estudios y Políticas Agrarias [ODEPA], 2018) and accumulates more than 50% of the Psa-infected orchards in Chile (**Figure 1** and **Table 1**). All of the isolates were confirmed as Psa strains by PCR using different sets of primers (see section “Materials and Methods”).

**FIGURE 2:**
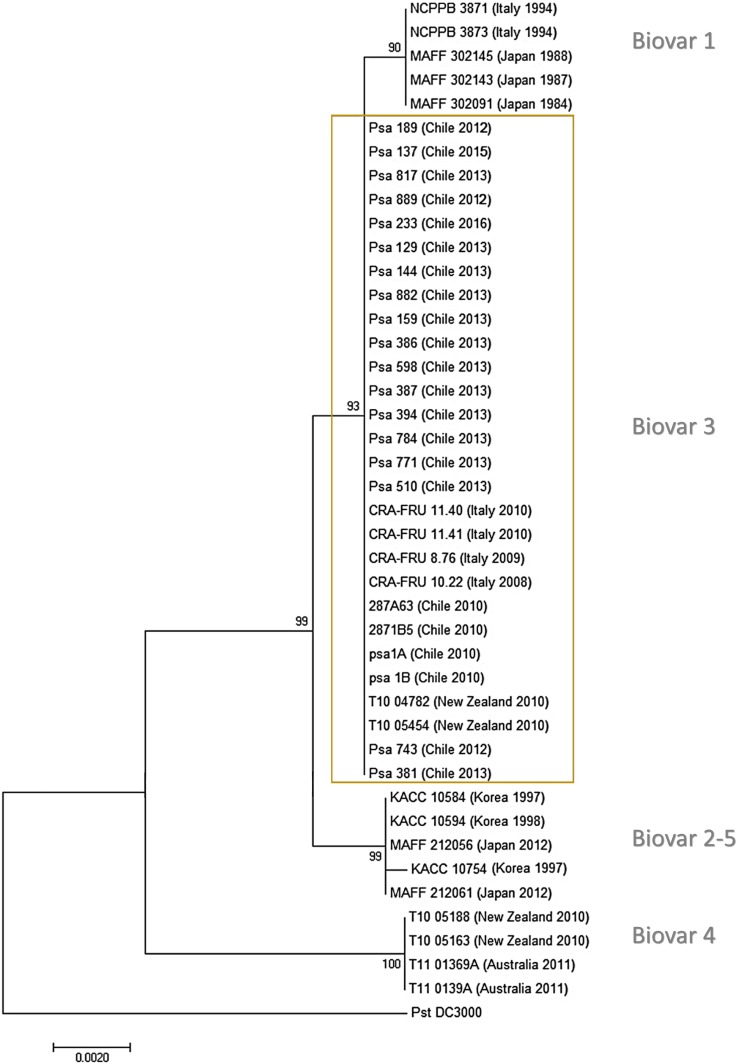
Phylogenetic tree of *Pseudomonas syringae* pv. *actinidiae* isolates derived from multilocus sequence analysis (MLSA). Phylogenetic tree using the neighbor-joining method (bootstrap: 1,000 replicates) and concatenated sequences of the genes *gapA, gltA, gyrB*, and *rpoB* for each isolate. Country, year of isolation, and biovar clade are indicated for each strain.

The first Chilean Psa isolates had been previously assigned to the biovar 3 group (Butler et al., 2013; McCann et al., 2013; Cunty et al., 2015a). An MLSA using the housekeeping genes *gyrB* (DNA gyrase B), *rpoD* (sigma factor 70), *gltA* (citrate synthase), and *gapA* (glyceraldehyde-3-phosphate dehydrogenase) showed that the genes sequenced have 100% identity with the corresponding genes in different Psa strains belonging to biovar 3, including Chilean strains obtained in 2010. The phylogenetic analysis including other Psa strains shows a clear clustering of different biovars except for biovar 2 and 5 that are grouped together (**Figure 2**). The results show that all the Chilean Psa isolates group together with the other Psa biovar 3 isolates, confirming the findings of previous studies. These results were also confirmed by the PCR detection of the 16 type III effector genes that have been described in Psa biovar 3 strains (McCann et al., 2013; Ferrante and Scortichini, 2015). Type III effector genes were detected in all of the Chilean Psa strains, including those encoded in plasmid DNA in Psa biovar 3. The identity of these genes was confirmed by sequencing the amplicons of Psa strain 743 as a representative of the other Chilean Psa strains (**Supplementary Table S2**). These results also suggest that no new biovars have been introduced to Chile during this period.

### Phenotypic Characterization of the Psa Isolates

Different features implicated in the fitness and virulence of Psa were evaluated in the 18 Chilean isolates. None of the strains showed differences in their growth parameters (data not shown). However, their biochemical profile determined using the Biolog GEN III MicroPlate revealed differences in 22% of the different tests in at least one of the 18 strains (**Supplementary Table S6**). All of the strains were able to use different carbon sources such as D-glucose, D-mannose, D-galactose, glycerol, D-mannitol, L-arginine, L-serine, acetic acid, and citric acid. However, they varied in their ability to use sucrose, D-fructose, inosine, L-glutamic acid, and formic acid. Alternatively, all of the strains were resistant to antibiotics such as rifamycin SV, lincomycin, and vancomycin, while they were sensitive to minocycline and troleandomycin and showed variable sensitivity to aztreonam, nalidixic acid, and fusidic acid. Despite these differences, all of the strains were identified as *P. syringae* pathovars according to the Biolog GEN III database (version 2.8). Interestingly, all of the isolates were susceptible to copper (MIC 75 μg/mL Cu^2+^) and streptomycin (MIC 3.9 μg/mL), suggesting that no resistance has developed in these strains despite the use of copper compounds as antimicrobials in the Chilean kiwifruit industry.

**FIGURE 3:**
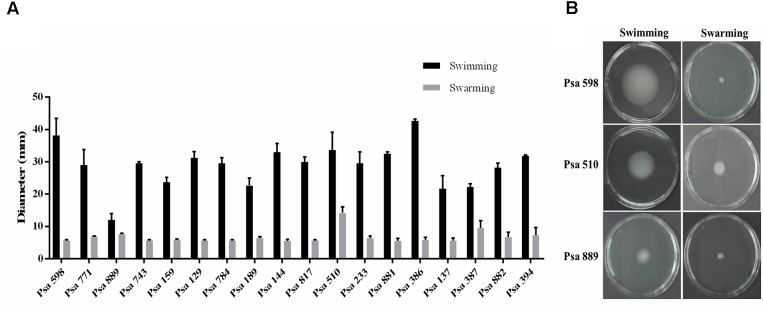
Motility of the Chilean Psa isolates. **(A)** Swimming or swarming motility of different Chilean Psa strains. **(B)** Representative image of selected strains. Swimming or swarming movement was determined at 72 h post-inoculation measuring the diameter of displacement. The names of each strain are shown.

Biofilm production has been proposed to be an important virulence factor in *P. syringae* (Ghods et al., 2015; Ueda and Saneoka, 2015). Therefore, the ability to produce biofilm was evaluated in the different Chilean Psa isolates. The results showed that none were able to produce biofilm over an abiotic surface. However, they do produce a thin layer of biofilm (pellicle) in the air–liquid interface. Initially a thin layer of cells was observed in the center of static cultures after 24 h of incubation, turning to a fully grown biofilm after 96 h (**Supplementary Figure S2**). Swimming and swarming motility was also evaluated among the different Psa isolates. The results show that all of the isolates exhibit swimming motility except for strain Psa 889 which shows a significant reduced displacement in comparison to the other strains (*p* < 0.05). In contrast, none of the strains except for Psa 510 demonstrated swarming motility under the experimental conditions (*p* < 0.05) (**Figure 3**). These results show that the Chilean Psa strains demonstrate a high phenotypic homogeneity with specific differences in particular strains.

### Indole Production in the Psa Isolates

**FIGURE 4:**
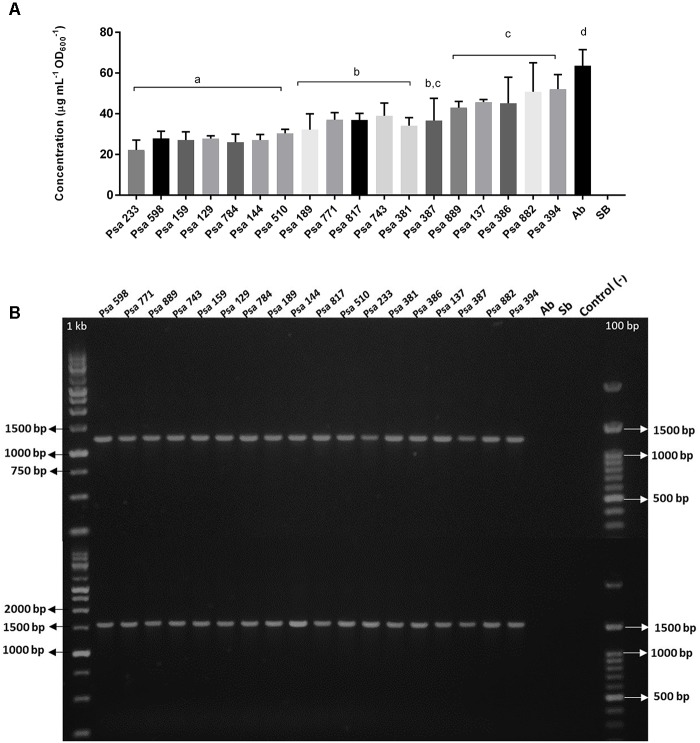
Indole production and detection of *iaaL/matE* genes in the Chilean Psa isolates. **(A)** Indole produced by the Chilean Psa isolates. Bacterial strains are organized according their level of indole production, and letters (a–d) show significant differences (*p* ≤ 0.05). The calibration curve with IAA (Sigma) is shown in **Supplementary Figure S3**. **(B)** PCR detection of genes *iaaL* (top) and *matE* (bottom). Molecular markers: 1 kb and 100 bp. Control (–): Reaction without DNA. *A. brasilense* (Ab) and *S. bongori* (Sb) were used for the positive and negative controls, respectively.

Indole-3-acetic acid production has been described in different *P. syringae* pathovars and *P. savastanoi* (Glickmann et al., 1998; Cerboneschi et al., 2016) but not in Psa. It is produced mostly from Trp via IAM by enzymes encoded in the genes *iaaM* and *iaaH*. Therefore, all 18 isolates were evaluated for their ability to produce IAA (Glickmann and Dessaux, 1995). The results show that all of the Chilean Psa isolates can produce indole at different concentrations (**Figure 4A**). In addition, some of the Chilean Psa isolates (Psa 882 and Psa 394) produce indole concentrations similar to those of *A. brasilense* (63 μg/mL IAA) that produces exceptionally large amounts of IAA (Bar and Okon, 1993). In all cases, indole was produced only in the presence of Trp, suggesting that, as observed in other *P. syringae*, this amino acid is the precursor of IAA synthesis in Psa. IAA production was also confirmed in the Chilean Psa strain 743 using LC-ESI-MS/MS analysis, showing a strong signal for IAA in the supernatant of the Psa 743 cell-free cultures (**Supplementary Figure S4**). The *iaaM* and *iaaH* genes were not detected in the Chilean Psa isolates using PCR and specific primers, suggesting an alternative route of synthesis exists in these strains. Recently, a novel IAA synthesis pathway was reported in *P. syringae* pv. *tomato* DC3000 (Pst), which involves the participation of an indole-3-acetaldehyde dehydrogenase encoded by the gene *aldA* and its homolog, *aldB* (McClerklin et al., 2018). Comparative analysis by BLASTN showed 95 and 97% identity between the *aldA* and *aldB* genes, and an aldehyde dehydrogenase sequence (GenBank accession number: CP011972.2: 149109–150602) and a carnitine dehydratase/3-oxoadipate enol-lactonase sequences (GenBank accession number: CP011972.2: 3182732–3184213) were encoded in the Biovar 3 Psa strain ICMP 18884. PCR with specific primers revealed that the *aldA* and *aldB* genes were also detected in all of the Chilean Psa strains, suggesting that they are likely to be responsible for the synthetic route of IAA. The identity of genes *aldA* and *aldB* was confirmed in strains Psa 889, Psa 743, and Psa 598 using Sanger sequencing (**Supplementary Table S2**).

It has also been reported that IAA can be conjugated to the amino acid lysine to produce IAA-Lys by the enzymatic activity of the *iaaL* gene product (Glickmann et al., 1998; Castillo-Lizardo et al., 2015; Cerboneschi et al., 2016). Our analysis detected the presence of the genes *iaaL* and *matE* in all of the Chilean Psa isolates (**Figure 4B**), which are in tandem in the Hrp regulon and are associated with IAA-Lys production. However, IAA-Lys production was not detected using the LC-ESI-MS/MS analysis. The identity of the *iaaL* and *matE* genes was also confirmed using Sanger sequencing in strains Psa 889, Psa 743, and Psa 598 (**Supplementary Table S3**). Taken together, these results strongly suggest that the Chilean Psa isolates produce IAA using a Trp-dependent pathway.

## Discussion

### Genetic Analysis of the Chilean Psa Isolates

*Pseudomonas syringae* pv. *actinidiae* was first isolated in Chile in 2010, and since then, it has been considered to be a quarantine pest under the official control of the SAG of Chile. The 18 Chilean Psa isolates included in this study were obtained as part of the monitoring program established by the SAG. They were isolated from the central south region of Chile, which is the zone that accumulates the majority of Psa infections reported in the country (Servicio Agrícola y Ganadero [SAG], 2018). These strains were obtained between the years 2012 and 2016, representing the most extended study performed on Psa in Chile. All of these strains were identified by the SAG and then confirmed by the standard molecular techniques used with this pathovar (Rees-George et al., 2010; Gallelli et al., 2011). As reported previously, the use of specific primers for the ITS amplification was not specific to Psa and also amplified a fragment from *P. syringae* (Vanneste, 2013). Therefore, a duplex-PCR analysis was necessary to positively identify the Psa isolates.

The MLSA confirmed that the Chilean Psa isolates belong to biovar 3. In this case, four housekeeping genes were used (*gyrB, rpoD, gltA*, and *gapA*), which seems to be sufficient to discriminate between biovar 3 and the other biovars; however, it is not sufficient to distinguish between biovars 2 and 5, which according to previous research, are very closely related (Fujikawa and Sawada, 2016). This phylogenetic analysis included sequences from several Psa strains with different biovars and origins, including some older Chilean strains that were also grouped in biovar 3. This suggests that this “hypervirulent” group (Ciarroni et al., 2015) is the only found in Chile, and no other biovar has entered or emerged. The conclusions of this study are consistent with previous research in which the Chilean Psa isolates were classified in the Psa Biovar 3 cluster using different approaches: REP-PCR fingerprinting, MLVA (multiple locus variable number of tandem repeats analysis) assay and MLST (Ferrante and Scortichini, 2010, 2015; Vanneste et al., 2010; Ciarroni et al., 2015; Biondi et al., 2017). Genomic analyses of the Chilean Psa strains suggest that they originated from China forming a sub-group in biovar 3 (Butler et al., 2013; Ciarroni et al., 2015).

Nearly 50 putative effector genes have been identified in Psa and are found in most of the biovars (McCann et al., 2013; Ferrante and Scortichini, 2015; Fujikawa and Sawada, 2016). Sixteen type III effector genes, among others, were identified in all of the Chilean Psa isolates, including genes that were reported in conjugative DNA plasmids in other biovar 3 Psa strains (*hopAV1* and *hopAU1*). The emergence of resistant strains as an evolutionary response to the use of antimicrobial compounds was observed in countries affected by recent outbreaks of Psa biovar 3 strains (Han et al., 2004; Vanneste, 2013; Colombi et al., 2017).

### Phenotypic Features of the Chilean Psa Isolates

The results of this study show a high phenotypic homogeneity. However, it is still possible to observe differences between specific features and specific strains. For instance, the biochemical profile shows differences between the various Chilean Psa strains (**Supplementary Table S6**). These differences are related to carbon source utilization and chemical susceptibility assays. Moura et al. (2015) reported similar results with different Psa isolates from Portugal. Using the BIOLOG system, they observed differences in the ability to use at least 12 different carbon sources among the Portuguese strains. Interestingly, both the Chilean and Portuguese strains varied in their ability to use methyl pyruvate, bromo-succinic acid, and acetoacetic acid as carbon sources showing that variations in the biochemical repertory are not exclusive to the Chilean strains. Both groups of strains are susceptible to minocycline, lithium chloride, and sodium butyrate. The Chilean Psa strains are also resistant to antibiotics not used in agriculture such as rifamycin SV or vancomycin. However, curiously they were susceptible to streptomycin (MIC 3.9 μg/mL) that, in the past, has been authorized for use to control Psa infections in Chile. This suggests that no resistance has evolved among the Chilean Psa strains, in contrast to what has been reported by others where Psa strains can have a MIC for streptomycin greater than 2,000 μg/mL (Cameron and Sarojini, 2014). A similar situation has been observed for copper resistance in which other studies have reported Psa strains with a MIC from 100 μg/mL to more than 1,000 μg/mL (Cameron and Sarojini, 2014), while the Chilean strains have a MIC of 75 μg/mL. The absence of resistance among the Chilean Psa strains could be due to multiple factors such as low selective pressures from the environment or low plasticity in the Psa genome of these strains. However, is not possible to disregard the existence of resistant Chilean Psa strains in the environment. Our results do not show a clear correlation between these differences in the biochemical profiles and the origin or isolation year of the strains, but it would be interesting to determine if these differences have any relevance for fitness or niche colonization in the natural environment of Psa.

All of the Chilean isolates demonstrate a similar range of swimming motility (**Figure 3**) with strain Psa 889 being the only exception that lacks motility. In contrast, none of the Chilean Psa strains show swarming motility, except for strain Psa 510 that demonstrates a slightly but significantly greater amount of displacement than the other strains. The differences observed between strains Psa 889 and Psa 510 are probably related to alterations in their flagella, since no differences were observed in the growth of any of the strains according to our analysis (**Supplementary Figure S5**). Flagellar motility is an important virulence factor that allows the infection of plants through natural openings on their tissue surfaces (Ichinose et al., 2013). Therefore, it remains to be determined if these differences in strains Psa 889 and Psa 510 are correlated with alterations in their virulence.

Psa infections are very persistent, and once they are detected in a region, it is very difficult or even impossible to eradicate the bacteria (Vanneste, 2017). This persistence could be related to the ability to endure environmental conditions through biofilm formation (Danhorn and Fuqua, 2007; Renzi et al., 2012). It has been reported that Psa can form biofilm (Ghods et al., 2015). However, our analysis showed that the Chilean Psa strains are not able to form biofilms over abiotic solid surfaces. This and other differences observed between the Chilean Psa strains and the other Psa are probably related to the unique clonal origin of the Psa strains present in Chile (Butler et al., 2013). However, the low affinity to form biofilms over solid surfaces has been observed in the *P. syringae* pathovars (Ueda and Saneoka, 2015). Therefore, it seems that biofilm formation is not a hallmark of this species. Interestingly the Chilean strains do form a thin layer of cells at the air–liquid interface in liquid cultures. This phenomenon has been described for other *Pseudomonas* species where an air–liquid interface would represent a favorable environment due to the oxygen access enabling a more rapid rate of growth (Constantin, 2009; Ueda and Saneoka, 2015). All of these results confirm the high degree of homogeneity among the different Chilean Psa strains. Further studies are needed to determine if the differences between the Chilean strains affect the colonization and infection of the kiwifruit plants.

### Indole Production in Psa Isolates

Several phytopathogens, including *P. syringae* pathovars, produce auxins that can alter the host’s physiology and promote plant susceptibility to infection (Glickmann et al., 1998; Cerboneschi et al., 2016). To our knowledge, this is the first report showing that Psa can produce indole using a Trp-dependent pathway. All of the Chilean Psa strains evaluated produce indole, some of them at levels similar to *A. brasilense*, which is a plant growth promoting bacterium (Masciarelli et al., 2013). The common route for IAA production in *P. syringae* pathovars is via the IAM pathway using the enzymes IaaM and IaaH. This pathway has been studied in *P. syringae* pv. *syringae* (Pss) and Psav (Glickmann et al., 1998; Baltrus et al., 2011; Aragón et al., 2014; Cerboneschi et al., 2016), and the only related report in Psa is from a strain isolated in 1984 belonging to biovar 1 which has putative ORFs of an IAM pathway (Baltrus et al., 2011). The Chilean Psa strains have the genes *aldA* and *aldB* which are associated with an alternative synthesis route of IAA recently found in *P. syringae* pv. *tomato* (McClerklin et al., 2018). Therefore, this is the most probable pathway in the Chilean Psa strains. Interestingly, bioinformatics analysis revealed that the genes *iaaH* and *iaaM*, associated with the common synthesis route of IAA, are only found in the Psa strains from biovar 4, which are now considered to be a new pathovar designated *P. syringae* pv. *actinidifoliorum* that is characterized by low virulence in kiwifruit plants (Abelleira et al., 2015). In this regard, the presence of the IAM pathway represents another distinctive feature differencing the former biovar 4 from the other Psa biovars.

*Pseudomonas syringae* pv. *tomato* and other species, such as *P. savastanoi* pv. *nerii*, also produce the enzyme IAA-lysine ligase, encoded by the *iaaL* gene, which is responsible for IAA-Lys production (Glickmann et al., 1998; Castillo-Lizardo et al., 2015; Cerboneschi et al., 2016). In the *P. syringae* pv. *tomato* (*Pst*) genome, *iaaL* is found in synteny with the *matE* gene that encodes a multidrug transporter of the MatE family. The analysis of the Chilean Psa strains revealed that all of the strains contain the genes *iaaL* and *matE*. A bioinformatic analysis showed that the *iaaL* gene was first annotated as a pre-protein translocase subunit Tim44 in several *P. syringae* pathovars; however, later it was annotated as an indoleacetate-lysine ligase gene in *P. syringae* pv. *tomato* (Castillo-Lizardo et al., 2015). According to this analysis, the *matE* and *iaaL* genes are conserved in Psa Biovar 1, 2, 3, and 5 strains with near 100% identity in their amino acid sequences (**Supplementary Figure S6**). There are reports on the importance of IAA production, and IAA-Lys in particular, in the virulence of *P. syringae*. For instance, mutations in the IAM pathway of Pss affect its growth in *Phaseolus vulgaris* (Mazzola and White, 1994), and the deletion of the *aldA, aldB, iaaL*, or *matE* genes in *P. syringae* pv. *tomato* result in a reduction in fitness, colonization, and virulence in infected tomato plants (Castillo-Lizardo et al., 2015; McClerklin et al., 2018). In addition, studies on the IAA-Lys effect on plants suggest that IAA conjugation can modulate hormone action and suppress the immune response (Romano et al., 1991). Our results show that the Chilean Psa strains produce IAA. However, we were not able to demonstrate IAA-Lys production. Despite this, the presence of the genes *iaaL* and *matE* in the Chilean and other Psa strains, including different biovars, raise the possibility that this compound could be produced in conditions other than those evaluated in this study. To date, the exact mechanism of action of IAA and IAA-Lys in the virulence of *P. syringae* species is not totally understood. The results presented here show that the Chilean Psa strains produce IAA, but it is unknown if this feature is shared with other Psa strains of biovar 3 and other biovars. The results represent the starting point to determine the mechanisms and regulation of IAA production (and possibly IAA-Lys) in Psa and its participation during infection in kiwifruits plants.

## Conclusion

The results of this study confirm that the Chilean Psa isolates belong to biovar 3. The isolates exhibit high homogeneity with phenotypic differences in specific isolates. This study is also the first report of Psa strains producing IAA using a Trp-dependent pathway. Several reports suggest that this compound may be related to virulence in *P. syringae* pathovars. Therefore, it would be interesting to determine whether this feature plays a role during bacterial canker in kiwi plants and to evaluate whether this is a common characteristic in different biovars of this pathovar.

## Author Contributions

OF, CY, XB, and RB conceived and designed the study, and analyzed the results. OF, CP, and MN performed the experiments. AV and CM performed the LC-ESI-MS/MS analysis. OF and RB wrote the manuscript. All authors reviewed and approved the final manuscript.

## Conflict of Interest Statement

The authors declare that the research was conducted in the absence of any commercial or financial relationships that could be construed as a potential conflict of interest.
