# Supplementary figures and images for: Genetic and Phenotypic Characterization of Indole-Producing Isolates of Pseudomonas syringae pv. actinidiae Obtained From Chilean Kiwifruit Orchards
Source: Front Microbiol. 2018 Aug 22;9:1907. doi: 10.3389/fmicb.2018.01907 (PMC6113925; doi:10.3389/fmicb.2018.01907)

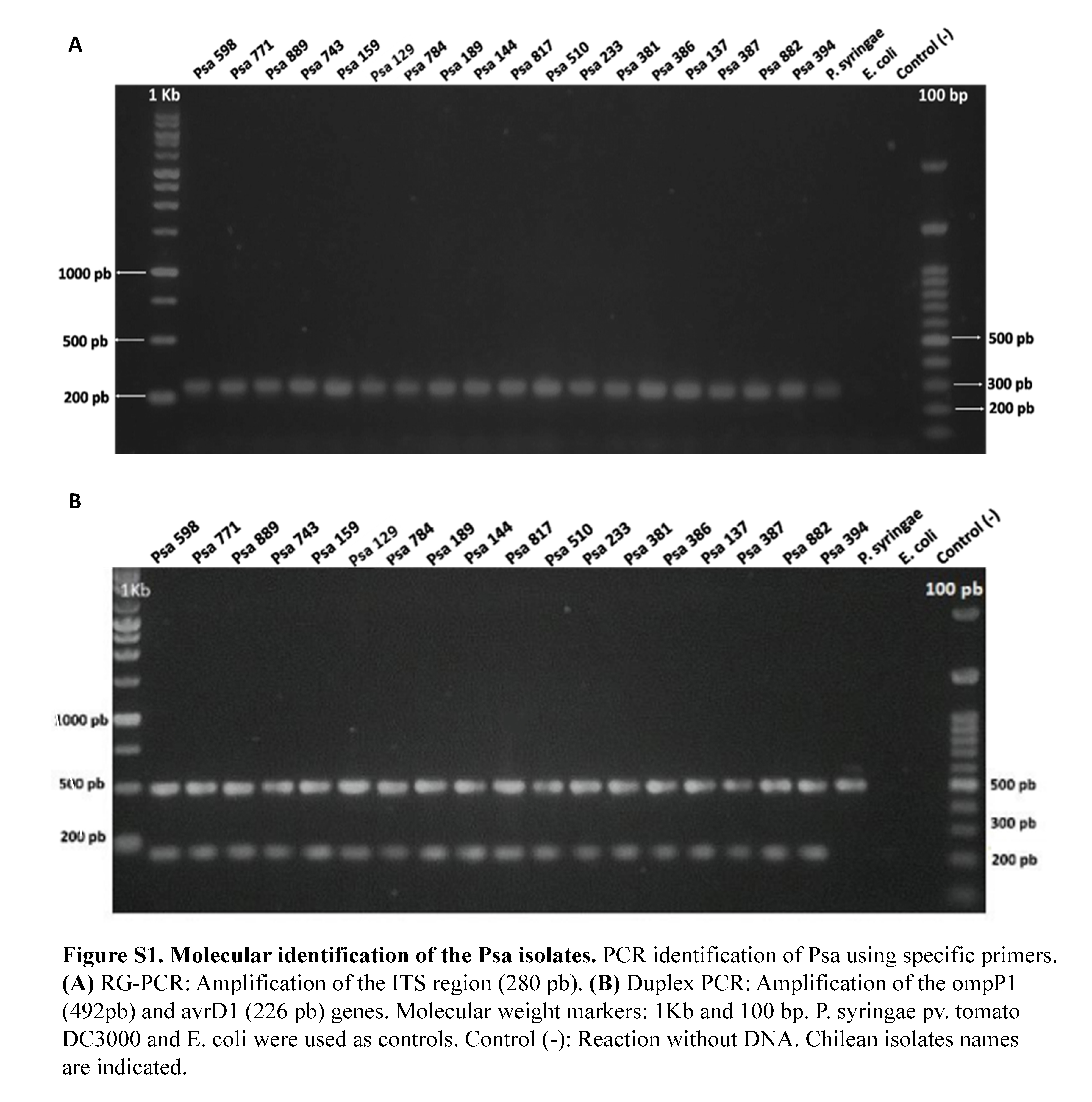

Supplement: Supplementary file 1 [file Image_1.JPEG]

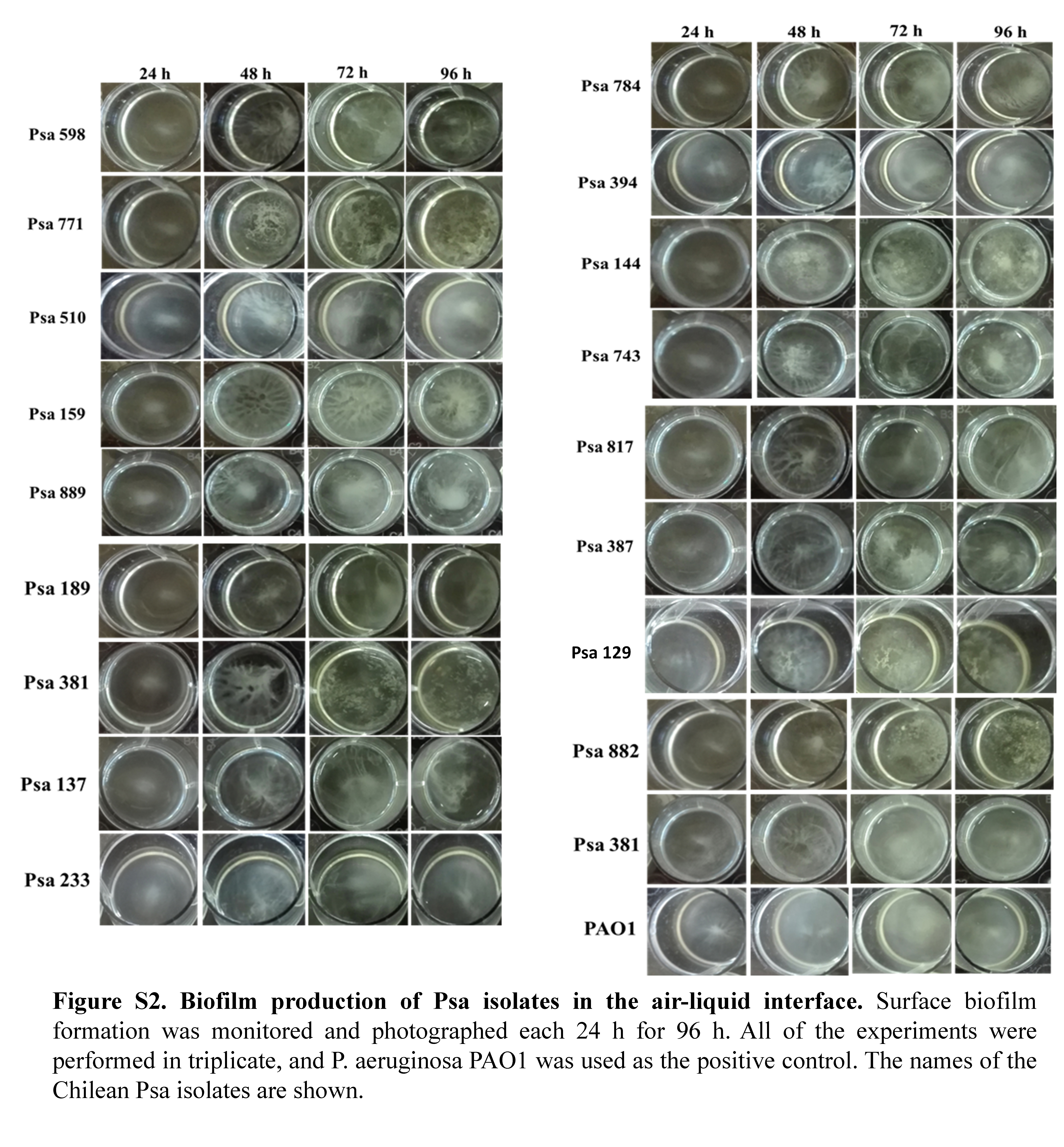

Supplement: Supplementary file 2 [file Image_2.JPEG]

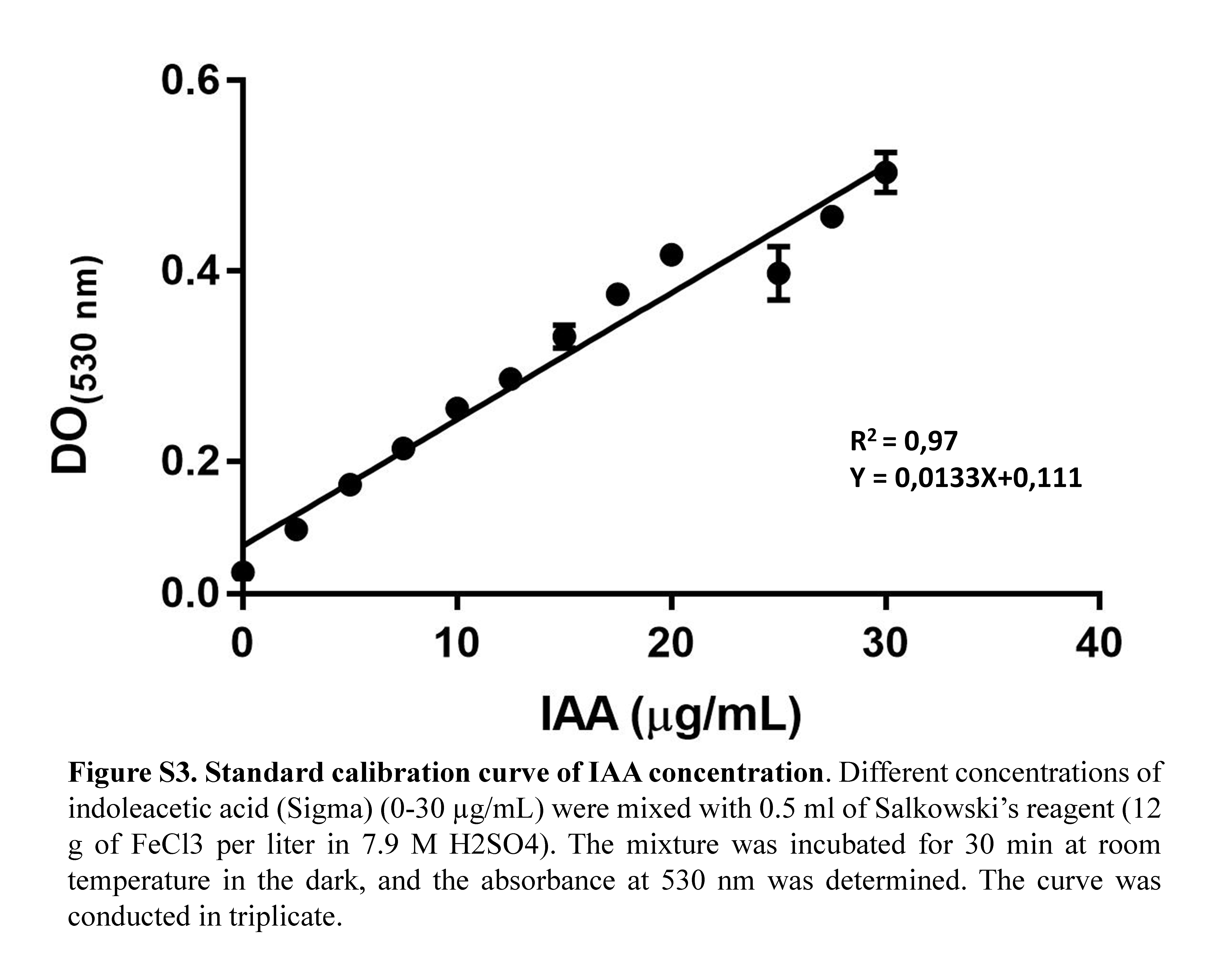

Supplement: Supplementary file 3 [file Image_3.JPEG]

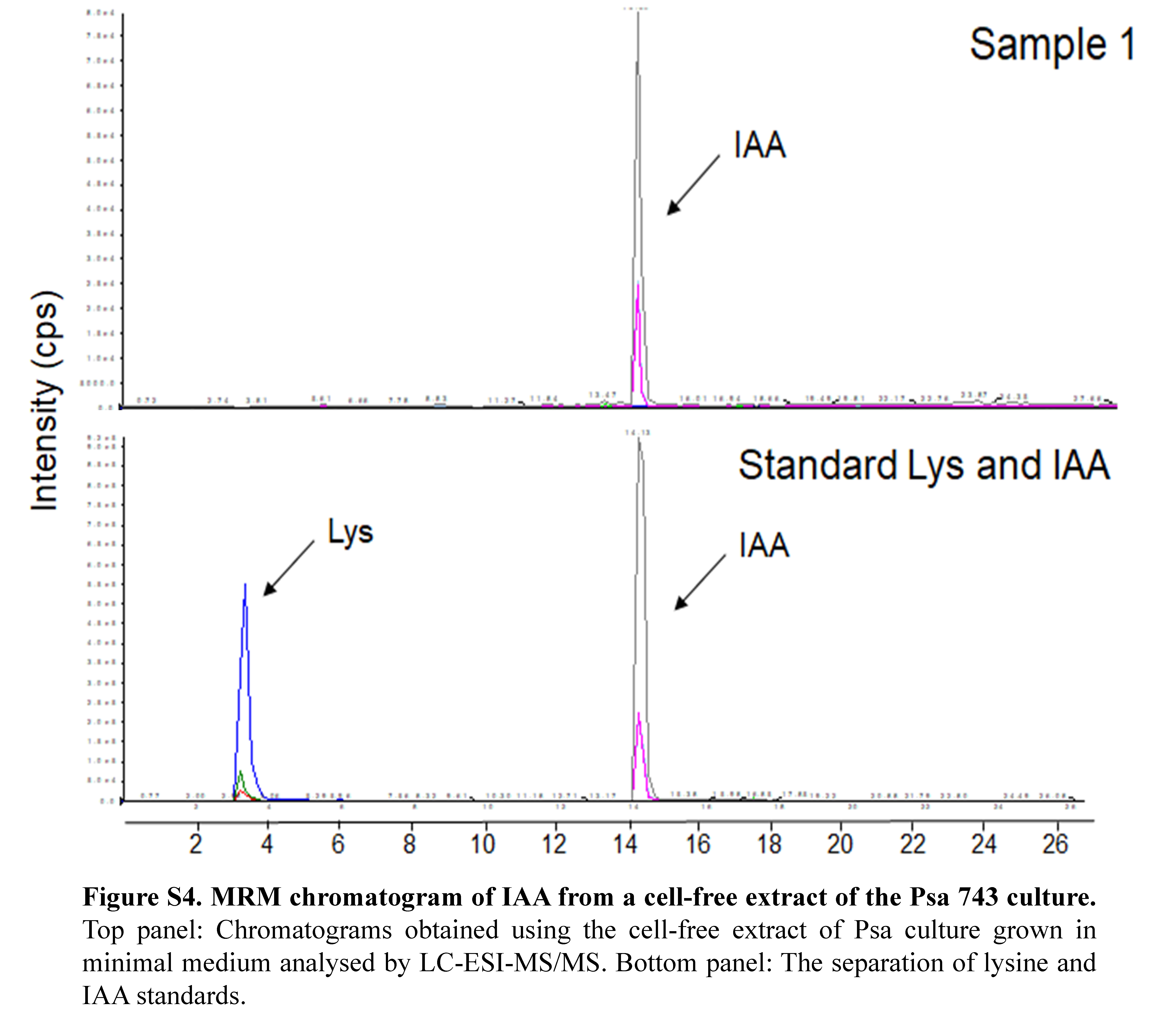

Supplement: Supplementary file 4 [file Image_4.JPEG]

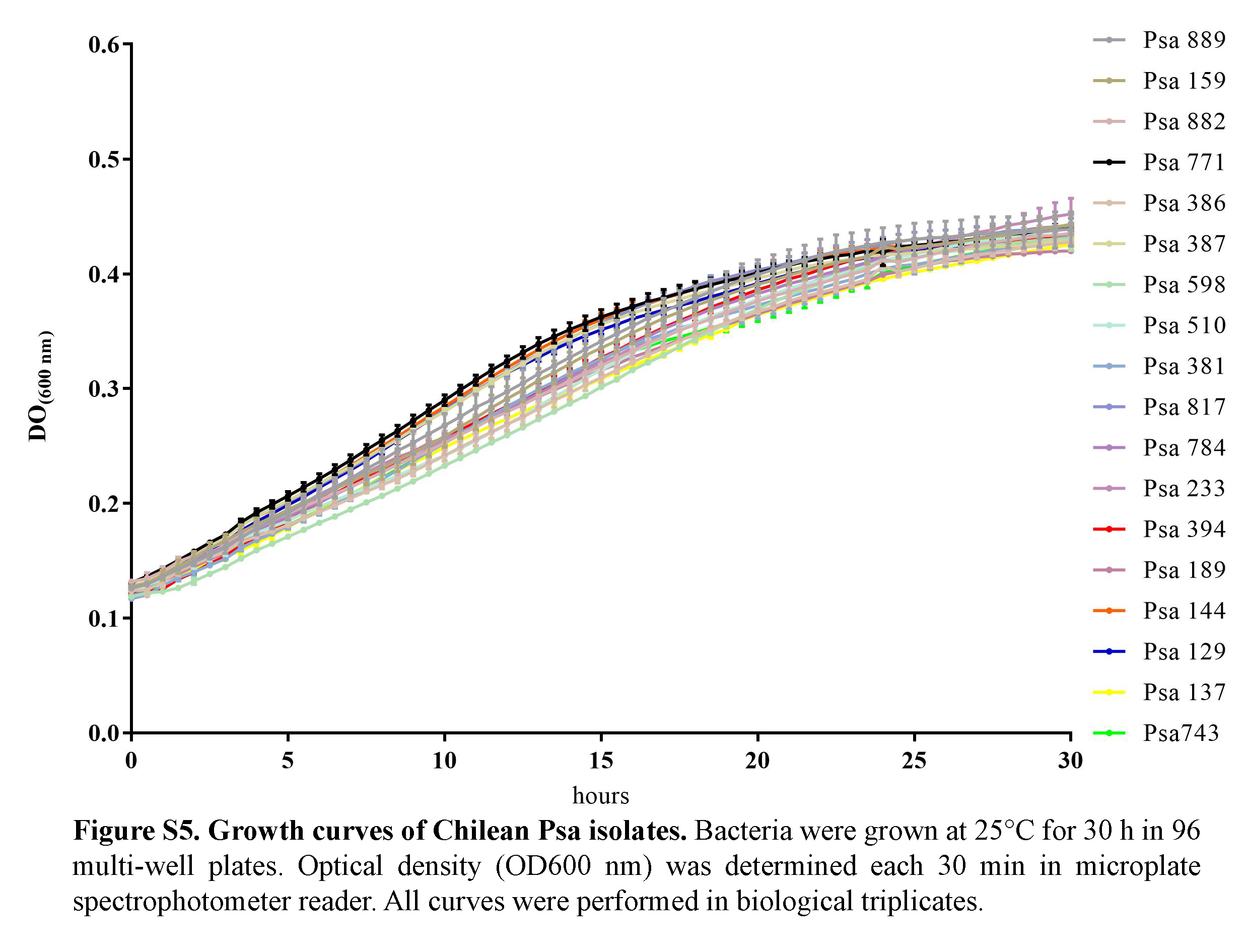

Supplement: Supplementary file 5 [file Image_5.JPEG]
