# Supplementary material for: Genetic and Phenotypic Characterization of Indole-Producing Isolates of Pseudomonas syringae pv. actinidiae Obtained From Chilean Kiwifruit Orchards
Source: Front Microbiol. 2018 Aug 22;9:1907. doi: 10.3389/fmicb.2018.01907 (PMC6113925; doi:10.3389/fmicb.2018.01907)
Supplement: Supplementary file 7 [file Table_1.DOCX]

**Table S1. Primers used in this study.**

| **PCR** | **Name** | **Primer sequence (5'-3')** | **Annealing T° (°C)** | **Reference** |
| --- | --- | --- | --- | --- |
| Psa identification by RG-PCR | PsaF1 | TTTTGCTTTGCACACCCGATTTT | 60 | Rees-George et al. (2010) |
|  | PsaR2 | CACGCACCCTTCAATCAGGATG |  |  |
| Psa identification by Duplex PCR | KNF-F | CACGGATACATGGGCTTATGC | 60 | Gallelli et al. (2011) |
|  | KNF-R | CTTTTCATCCACACACTCCG |  |  |
|  | AvrD1-F | TTTCGGTGGTAACGTTGGCA |  |  |
|  | AvrD1-R | TTCCGCTAGGTGAAAAATGGG |  |  |
| MLSA | GapA-F | CGCCATYCGCAACCCG | 60 | Ferrante and Scortichini (2010) |
|  | GapA-R | CCCAYTCGTTGTCGTACCA |  |  |
|  | GyrB-F | MGGCGGYAAGTTCGATGACAAYTC |  |  |
|  | GyrB-R | TRATBKCAGTCARACCTTCRCGSGC |  |  |
|  | GltA-F | AGTTGATCATCGAGGGCGCWGCC |  |  |
|  | GltA-R | TGATCGGTTTGATCTCGCACGG |  |  |
|  | RpoD-F | AAGGCGARATCGAAATCGCCAAGCG | 65 |  |
|  | RpoD-R | GGAACWKGCGCAGGAGTCGGCACG |  |  |
| Detection of *mate-iaaL* operon | iaaL-F1 | GGACTCTCATGACTGCCTAC | 60 | This study |
|  | iaaL-R2 | GGCGGTCAATGATGTAGAGC |  |  |
|  | matE-F1 | TGGCGGTTACCAAAGCAGTC | 64 |  |
|  | matE-R2 | CGTAGGCAGTCATGAGAGTC |  |  |
| Detection of Indole-3-acetamida (IAM) pathway | iaaH-F | CAGATTGCCGCTGAAGAGCT | 55-65* |  |
|  | iaaH-R | CCAGATCAGACCACATGGAG |  |  |
|  | iaaM-F | GTACGACTACGGTCCCTTTC |  |  |
|  | iaaM-R | GATATCCCGTCCGGAATCAG |  |  |
| Detection of IAA synthesis pathway | aldAF | ACCTTTGACTGCATCAGCCC | 60 |  |
|  | aldAR | ATTTGTCGAACGCGTGCAGC |  |  |
|  | aldBF | ATGAGCCTTACCCGCTTTCAG |  |  |
|  | aldBR | GACAAATGGATCAGGCATGGGC |  |  |
| Detection of type III secretion system effector genes | avrE1F | ACTCAGACAACCCGATGACC | 65 | Ferrante and Scotichini (2015) |
|  | avrE1R | TCACCGTCGTGACTGAGAAG |  |  |
|  | hopA1F | CCATTCAGTCACGCTTCTCC | 65 | This study |
|  | hopA1R | GAAAAACGCCGGTCCTGGGT |  |  |
|  | hopF2F | CTCATCATGTGTACAGCCCC |  |  |
|  | hopF2R | GCACCTTACGCCCTTTTGGC |  |  |
|  | hopH1F | GCATCTATATCGCCTCCCTC |  |  |
|  | hopH1R | TGGCCTGTACTTCATGCGCAG |  |  |
|  | hopM1F | GGACGGGCTGAATGAAAGTA | 55 | Ferrante and Scotichini (2015) |
|  | hopM1R | TCATTTCCTGAGCGGTTTTC |  |  |
|  | hopZ5F | GCATCAAAACCCCGCACAAC | 65 | This study |
|  | hopZ5R | CGCTCACTTCTTTGAACTCG |  |  |
|  | hopAA1-1F | ATGCACATCAACCGACCCGTC | 67 |  |
|  | hopAA1-1R | TTACGAGCGCATAGGCCGAAAC |  |  |
|  | hopAF1F | AGCAGAAAGACGGCACAGTG | 65 |  |
|  | hopAF1R | ATAACAGCGCCCAGCAGAGTG |  |  |
|  | hopAM1-1F | TGCCGCAACTCATTATCTTG | 65 | Ferrante and Scotichini (2015) |
|  | hopAM1-1R | TTTCGCTAAGCCACTGGAAT |  |  |
|  | hopAO2F | TCATTACTTCCCGAGGCGTC | 65 | This study |
|  | hopAO2R | AAGCGTTGTTGAGAGGGGGTG |  |  |
|  | hopAU1F | CGAGATACCGACACGCATGA | 66 |  |
|  | hopAU1R | CAACGCATGCTGACTATGGC |  |  |
|  | hopAV1F | GACGTTACGAAGATGCACGC | 66 |  |
|  | hopAV1R | GACACGTACAGACGCCCAAG |  |  |
|  | hopAW1F | CAACCAGCGGTCGAACATAC | 65 |  |
|  | hopAW1R | GAGCGCACAGGCAGAATATG |  |  |
|  | hopBB1-F | TTGTGGTACTTCCGGCTCTC |  |  |
|  | hopBB1-R | AAGGTGAACGTCGGTATGGC |  |  |
|  | hopBNF | GTCCGTCCAGAGTTACGATG |  |  |
|  | hopBNR | TGCCATCCAGAACCAGAGTC |  |  |

*Annealing temperature gradient.

**Table S2. Sequenced genes in Chilean Psa isolates.**

| **Gene name** | **Protein name** | **Function in *P. syringae* pathovars** | **Accession number NCBI^a^** |
| --- | --- | --- | --- |
| *avrE1* | AvrE1 |  | MH464116 |
| *hopA1* | HopA1 |  | MH464117 |
| *hopF2* | HopF2 |  | MH464112 |
| *hopH1* | HopH1 |  | MH464113 |
| *hopM1* | HopM1 |  | MH464114 |
| *hopAA1-1* | HopAA1-1 |  | MH464118 |
| *hopAF1* | HopAF1 |  | MH464119 |
| *hopZ5* | HopZ5 | Type III secretion | MH464115 |
| *hopAM1-1* | HopAM1-1 | system effector proteins | MH464120 |
| *HopAW1* | HopAW1 |  | MH464124 |
| *hopAO2* | HopAO2 |  | MH464121 |
| *hopBB1^b^* | HopBB1-1 |  | MH464110 |
|  | HopBB1-2 |  |  |
| *hopBN1* | HopBN1 |  | MH464111 |
| *hopAU1* | HopAU1 |  | MH464122 |
| *hopAV1* | HopAV1 |  | MH464123 |
| *iaaL* | Indoleacetic acid-lysine synthetase | Synthesis of IAA-L | MH464125, MH464126, MH464128 |
| *mate* | MATE family transporter | IAA transport | MH464129, MH464127, MH464130 |
| *aldA* | Indole-3-acetaldehyde dehydrogenase | IAA synthesis | MH464131, MH464133, MH464132 |
| *aldB* | Aldehyde dehydrogenase family protein (homologue to AldA) | IAA synthesis | MH464131, MH464133, MH464132 |

^a^Accession numbers of *iaaL*, *matE*, *aldA*, *aldB* genes belongs to Psa889, Psa598 and Psa743, respectively**.** Accession numbers of Type III secretion system effector genes are from Psa 743.

^b^ Genes *HopBB1-1* and *HopBB1-2* are homologues.

**Table S3. Accession numbers of sequences obtained from Chilean Psa strains for MLSA.**

| **Isolate** | **Accession number* *gapA/gltA/gyrB/rpoD*** |
| --- | --- |
| Psa 743 | MF547625 / MF547652 / MF547607 / MF547589 |
| Psa 889 | MF547640 / MF547649 / MF547622 / MF547604 |
| Psa 817 | MF547641 / MF547650 / MF547623 / MF547605 |
| Psa 381 | MF547626 / MF547653 / MF547608 / MF547590 |
| Psa 510 | MF547627 / MF547654 / MF547609 / MF547591 |
| Psa 771 | MF547628 / MF547655 / MF547610 / MF547592 |
| Psa 784 | MF547629 / MF547656 / MF547611 / MF547593 |
| Psa 394 | MF547630 / MF547657 / MF547612 / MF547594 |
| Psa 387 | MF547631 / MF547658 / MF547613 / MF547595 |
| Psa 882 | MF547636 / MF547645 / MF547618 / MF547600 |
| Psa 144 | MF547637 / MF547646 / MF547619 / MF547601 |
| Psa 598 | MF547632 / MF547659 / MF547614 / MF547596 |
| Psa 386 | MF547633 / MF547660 / MF547615 / MF547597 |
| Psa 159 | MF547634 / MF547643 / MF547616 / MF547598 |
| Psa 189 | MF547635 / MF547644 / MF547617 / MF547599 |
| Psa 129 | MF547638 / MF547647 / MF547620 / MF547602 |
| Psa 137 | MF547642 / MF547651 / MF547624 / MF547606 |
| Psa 233 | MF547639 / MF547648 / MF547621 / MF547603 |

**Table S4. Sequences of Psa Biovars used in MLSA analysis**

| **Biovar** | **Strain** | **Origin** | **year** | **Accession number NCBI: *gapA* / cts (*gltA*) / *gyrB*/ *rpoD*** |
| --- | --- | --- | --- | --- |
|  | MAFF 302145 | Japan | 1988 | JN683491.1 / JN683510.1 / JN683472.1 / JN683415.1 |
|  | MAFF 302091 | Japan | 1984 | JN683489.1 / JN683508.1 / JN683470.1 / JN683413.1 |
| 1 | MAFF 302143 | Japan | 1987 | JN683490.1 / JN683509.1 / JN683471.1 / JN683414.1 |
|  | NCPPB 3871 | Italy | 1994 | KF937495.1 / KF937495.1 / KF937689.1 / KF937786.1 |
|  | NCPPB 3873 | Italy | 1994 | JX288012.1 / JX287995.1 / JX288029.1 / JX287912.1 |
|  | KACC 10754 | Korea | 1997 | JN683488.1 / JN683507.1 / JN683469.1 / JN683412.1 |
| 2 | KACC 10584 | Korea | 1997 | JN683486.1 / JN683505.1 / JN683467.1 / JN683410.1 |
|  | KACC 10594 | Korea | 1998 | JN683487.1 / JN683506.1 / JN683468.1 / JN683411.1 |
|  | CRA-FRU 8.76 | Italy | 2009 | JX288015.1 / JX287998.1 / JX288032.1 / JX287915.1 |
|  | CRA-FRU 10.22 | Italy | 2008 | JX288014.1 / JX287997.1 / JX288031.1 / JX287914.1 |
|  | CRA-FRU 11.40 | Italy | 2010 | JX288016.1 / JX287999.1 / JX288033.1 / JX287916.1 |
|  | CRA-FRU 11.41 | Italy | 2010 | JX288017.1 / JX288000.1 / JX288034.1 / JX287917.1 |
| 3 | T10_04782 | New Zealand | 2010 | JN683475.1 / JN683494.1 / JN683456.1 / JN683399.1 |
|  | T10_05454 | New Zealand | 2010 | JN683476.1 / JN683495.1 / JN683457.1 / JN683400.1 |
|  | Psa1A | Chile | 2010 | JQ836620.1 / JQ836616.1 / JQ836624.1 / JQ836579.1 |
|  | Psa1B | Chile | 2010 | JQ836621.1 / JQ836617.1 / JQ836625.1 / JQ836580.1 |
|  | 287A63 | Chile | 2010 | JX288007.1 / JX287990.1 / JX288024.1 / JX287907.1 |
|  | 2871B5 | Chile | 2010 | JX288009.1 / JX287992.1 / JX288026.1 / JX287909.1 |
|  | T10_05163 | New Zealand | 2010 | JN683480.1 / JN683499.1 / JN683461.1 / JN683404.1 |
|  | T10_05188 | New Zealand | 2010 | JN683481.1 / JN683500.1 / JN683462.1 / JN683405.1 |
| 4 | T11_01369A | Australia | 2011 | JQ836622.1 / JQ836618.1 / JQ836626.1 / JQ836581.1 |
|  | T11_01395ª | Australia | 2011 | JQ836623.1 / JQ836619.1 / JQ836627.1 / JQ836582.1 |
|  | CFBP7951 | France | 2010 | KF937418.1 / KF937520.1 / KF937612.1 / KF937714.1 |
| 5 | MAFF 212056 | Japan | 2012 | AB842211.1 / AB842199.1 / AB842223.1 / AB842259.1 |
|  | MAFF 212061 | Japan | 2012 | AB842216.1 / AB842204.1 / AB842228.1 / AB842264.1 |

**Table S5. Sequences of indole pathway genes used in this study**

| **Gene** | **Strain** | **Accession number Genbank (NCBI)** |
| --- | --- | --- |
| *iaaH / iaaM* | *P. syringae pv. actinidiae* ICMP 19497 (Biovar 4) | NZ_LKBQ01000038.1:3594-4934 / LKBQ01000038.1:1884-3557 |
|  | *P. syringae pv. actinidiae* ICMP 19100 (Biovar 4) | NZ_AOKC01000520.1:3649-4989 / NZ_AOKC01000520.1:1939-3612 |
|  | *P. syringae pv. actinidiae* ICMP 19098 ((Biovar 4) | NZ_AOKE01000107.1:33987-35327 / NZ_AOKE01000107.1:32277-33950 |
|  | *P. syringae pv. actinidiae* ICMP 19095 (Biovar 4) | NZ_AOKI01000721.1:3587-4927 / NZ_AOKI01000721.1:1877-3550 |
|  | *P. syringae pv. actinidiae* ICMP 19094 (Biovar 4) | NZ_AOKJ01000444.1:c3251-1911 / NZ_AOKJ01000444.1:c4961-3288 |
|  | *P. syringae pv. actinidiae* ICMP 18807 (Biovar 4) | NZ_ANJL01000105.1:c3464-2124 / NZ_ANJL01000105.1:3501-5174 |
|  | *P. syringae pv. actinidiae* ICMP 18804 (Biovar 4) | NZ_ANJE01000191.1:c3763-2423 / NZ_ANJE01000191.1:3800-5194 |
|  | *P. syringae pv. actinidiae* ICMP 18883 (Biovar 4) | NZ_AOKH01000154.1:29795-31135/NZ_AOKH01000154.1:28085-29758 |
|  | *P. syringae pv. syringae* Y30 | U04358.1 |
|  | *P. syringae pv. syringae* B728a | CP000075.1:1727496-1728836 / CP000075.1:1725786-1727459 |
| *matE* / *iaaL* | *P. syringae pv. actinidiae* NZ-45 (Biovar 3) | CP017007.1:421984-423327 / CP017007.1:423442-424629 |
|  | *P. syringae pv. actinidiae* CRAFRU 14.08 (Biovar 3) | CP019732.1:421981-423356 / CP019732.1:423439-424626 |
|  | *P. syringae pv. actinidiae* CRAFRU 12.29 (Biovar 3) | CP019730.1:421981-423356 / CP019730.1:423439-424626 |
|  | *P. syringae pv. actinidiae* ICMP 18884 (Biovar 3) | CP011972.2:421983-423326/ CP011972.2:423441-424628 |
|  | *P. syringae pv. actinidiae* ICMP 18708 (Biovar 3) | CP012179.1:421984-423327 / CP012179.1:423442-424629 |
|  | *P. syringae pv. actinidiae* NZ-47 (Biovar 3) | CP017009.1:421983-423326/ CP017009.1:423441-424628 |
|  | *P. syringae pv. actinidiae* ICMP 9853 (Biovar 1) | CP018202.1:6311081-6312424/ CP018202.1:6309779-6310966 |
|  | *P. syringae pv. tomato* DC3000 | NC_004578.1: 404865-406241 / NC_004578.1: 406323-407510 |
| *adlA /adlB* | *P. syringae pv. tomato* DC3000 | NC_004578.1: 111972-113465/ NC_004578.1:c2968411-2969892 |

**Table S6. Biochemical pattern obtained for Chilean Psa isolates by BIOLOG GEN III**

|  | **Chilean Psa strains^b^** | | | | | | | | | | | | | | | | | |
| --- | --- | --- | --- | --- | --- | --- | --- | --- | --- | --- | --- | --- | --- | --- | --- | --- | --- | --- |
| **Sustrate^a^** | **159** | **817** | **189** | **137** | **743** | **889** | **598** | **233** | **381** | **882** | **129** | **784** | **386** | **510** | **387** | **144** | **771** | **394** |
| **Sucrose** |  |  |  |  |  |  |  |  |  |  |  |  |  |  |  |  |  |  |
| **Neuraminic Acid** |  |  |  |  |  |  |  |  |  |  |  |  |  |  |  |  |  |  |
| **D-Glucose** |  |  |  |  |  |  |  |  |  |  |  |  |  |  |  |  |  |  |
| **D-Mannose** |  |  |  |  |  |  |  |  |  |  |  |  |  |  |  |  |  |  |
| **D-Fructose** |  |  |  |  |  |  |  |  |  |  |  |  |  |  |  |  |  |  |
| **D-Galactose** |  |  |  |  |  |  |  |  |  |  |  |  |  |  |  |  |  |  |
| **Inosine** |  |  |  |  |  |  |  |  |  |  |  |  |  |  |  |  |  |  |
| **D-Sorbitol** |  |  |  |  |  |  |  |  |  |  |  |  |  |  |  |  |  |  |
| **D-Mannitol** |  |  |  |  |  |  |  |  |  |  |  |  |  |  |  |  |  |  |
| **D-Arabitol** |  |  |  |  |  |  |  |  |  |  |  |  |  |  |  |  |  |  |
| **myo-Inositol** |  |  |  |  |  |  |  |  |  |  |  |  |  |  |  |  |  |  |
| **Glycerol** |  |  |  |  |  |  |  |  |  |  |  |  |  |  |  |  |  |  |
| **L-Arginine** |  |  |  |  |  |  |  |  |  |  |  |  |  |  |  |  |  |  |
| **L-Aspartic Acid** |  |  |  |  |  |  |  |  |  |  |  |  |  |  |  |  |  |  |
| **L-Glutamic Acid** |  |  |  |  |  |  |  |  |  |  |  |  |  |  |  |  |  |  |
| **L-Pyroglutamic Acid** |  |  |  |  |  |  |  |  |  |  |  |  |  |  |  |  |  |  |
| **L-Serine** |  |  |  |  |  |  |  |  |  |  |  |  |  |  |  |  |  |  |
| **D-Gluconic Acid** |  |  |  |  |  |  |  |  |  |  |  |  |  |  |  |  |  |  |
| **D-Glucuronic Acid** |  |  |  |  |  |  |  |  |  |  |  |  |  |  |  |  |  |  |
| **Mucic Acid** |  |  |  |  |  |  |  |  |  |  |  |  |  |  |  |  |  |  |
| **Quinic Acid** |  |  |  |  |  |  |  |  |  |  |  |  |  |  |  |  |  |  |
| **D-Saccharic Acid** |  |  |  |  |  |  |  |  |  |  |  |  |  |  |  |  |  |  |
| **Citric Acid** |  |  |  |  |  |  |  |  |  |  |  |  |  |  |  |  |  |  |
| **D-Malic Acid** |  |  |  |  |  |  |  |  |  |  |  |  |  |  |  |  |  |  |
| **L-Malic Acid** |  |  |  |  |  |  |  |  |  |  |  |  |  |  |  |  |  |  |
| **-Amino-Butryric Acid** |  |  |  |  |  |  |  |  |  |  |  |  |  |  |  |  |  |  |
| **Propionic Acid** |  |  |  |  |  |  |  |  |  |  |  |  |  |  |  |  |  |  |
| **Acetic Acid** |  |  |  |  |  |  |  |  |  |  |  |  |  |  |  |  |  |  |
| **Formic Acid** |  |  |  |  |  |  |  |  |  |  |  |  |  |  |  |  |  |  |
| **pH 6** |  |  |  |  |  |  |  |  |  |  |  |  |  |  |  |  |  |  |
| **pH 5** |  |  |  |  |  |  |  |  |  |  |  |  |  |  |  |  |  |  |
| **1% Sodium lactate** |  |  |  |  |  |  |  |  |  |  |  |  |  |  |  |  |  |  |
| **Rifamycin SV** |  |  |  |  |  |  |  |  |  |  |  |  |  |  |  |  |  |  |
| **Lincomycin** |  |  |  |  |  |  |  |  |  |  |  |  |  |  |  |  |  |  |
| **Niaproff4** |  |  |  |  |  |  |  |  |  |  |  |  |  |  |  |  |  |  |
| **Vancomycin** |  |  |  |  |  |  |  |  |  |  |  |  |  |  |  |  |  |  |
| **Tetrazolium Violet** |  |  |  |  |  |  |  |  |  |  |  |  |  |  |  |  |  |  |
| **Tetrazolium Blue** |  |  |  |  |  |  |  |  |  |  |  |  |  |  |  |  |  |  |
| **Potassium Tellurite** |  |  |  |  |  |  |  |  |  |  |  |  |  |  |  |  |  |  |
| **Sodium Bromate** |  |  |  |  |  |  |  |  |  |  |  |  |  |  |  |  |  |  |
| **Glycyl-L-Proline** |  |  |  |  |  |  |  |  |  |  |  |  |  |  |  |  |  |  |
| **L-Alanine** |  |  |  |  |  |  |  |  |  |  |  |  |  |  |  |  |  |  |
| **L-Galactonic Acid Lactone** |  |  |  |  |  |  |  |  |  |  |  |  |  |  |  |  |  |  |
| **Methyl Pyruvate** |  |  |  |  |  |  |  |  |  |  |  |  |  |  |  |  |  |  |
| **L-Lactic Acid** |  |  |  |  |  |  |  |  |  |  |  |  |  |  |  |  |  |  |
| **Tween 40** |  |  |  |  |  |  |  |  |  |  |  |  |  |  |  |  |  |  |
| **β-Hydroxy-D,L-Butyric Acid** |  |  |  |  |  |  |  |  |  |  |  |  |  |  |  |  |  |  |
| **1% NaCl** |  |  |  |  |  |  |  |  |  |  |  |  |  |  |  |  |  |  |
| **Fusidic Acid** |  |  |  |  |  |  |  |  |  |  |  |  |  |  |  |  |  |  |
| **Nalidixic Acid** |  |  |  |  |  |  |  |  |  |  |  |  |  |  |  |  |  |  |
| **D-Serine** |  |  |  |  |  |  |  |  |  |  |  |  |  |  |  |  |  |  |
| **p-Hydroxy-Phenylacetic Acid** |  |  |  |  |  |  |  |  |  |  |  |  |  |  |  |  |  |  |
| **Bromo-Succinic Acid** |  |  |  |  |  |  |  |  |  |  |  |  |  |  |  |  |  |  |
| **Lithium Chloride** |  |  |  |  |  |  |  |  |  |  |  |  |  |  |  |  |  |  |
| **Aztreonam** |  |  |  |  |  |  |  |  |  |  |  |  |  |  |  |  |  |  |

1. Substrates: Red: Chemical susceptibly assay / Black: Carbon source utilization assay
2. Color indicates positive (Violet), negative (white) or borderline (light violet).
